# Effect of citalopram and sertraline on the expression of miRNA- 124, 132, and 16 and their protein targets in patients with depression

**DOI:** 10.22038/IJBMS.2023.66496.14595

**Published:** 2023

**Authors:** Mahnaz Ahmadimanesh, Leila Etemad, Dorsa Morshedi Rad, Mohammad Hossein Ghahremani, Amir Hooshang Mohammadpour, Reza Jafarzadeh Esfehani, Paul Jowsey, Fatemeh Behdani, Seyed Adel Moallem, Mohammad Reza Abbaszadegan

**Affiliations:** 1 Department of Pharmacodynamics and Toxicology, School of Pharmacy, Mashhad University of Medical Sciences, Mashhad, Iran; 2 Food and Drug Vice Presidency, Mashhad University of Medical Sciences, Mashhad, Iran; 3 Pharmaceutical Research Center, Pharmaceutical Technology Institute, Mashhad University of Medical Sciences, Mashhad, Iran; 4 International UNESCO Center for Health-Related Basic Sciences and Human Nutrition, Faculty of Medicine, Mashhad University of Medical Sciences, Mashhad, Iran; 5 Center of Health Technologies, School of Biomedical Engineering, University of Technology Sydney, Sydney, NSW 2007, Australia; 6 Department of Toxicology-Pharmacology, Faculty of Pharmacy, Tehran University of Medical Sciences, Tehran, Iran; 7 Department of clinical pharmacy, School of Pharmacy, Mashhad University of Medical Sciences, Mashhad, Iran; 8 Department of Medical Genetics, Faculty of Medicine, Mashhad University of Medical Sciences, Mashhad, Iran; 9 Medical Genetics Research Center, Basic Medical Sciences Institute, Mashhad University of Medical Sciences, Mashhad, Iran; 10 National Institute for Health Research (NIHR), Health Protection Research Unit for Chemical and Radiation Threats and Hazards, Institute of Cellular Medicine, Newcastle University, Newcastle Upon Tyne, UK; 11 Psychiatry and Behavioral Sciences Research Center, School of Medicine, Mashhad University of Medical Sciences, Mashhad, Iran; 12 Department of Pharmacology and Toxicology, College of Pharmacy, Al-Zahraa University for Women, Karbala, Iraq

**Keywords:** Citalopram, Depression, MiRNAs, Sertraline, SSRIs

## Abstract

**Objective(s):**

This study aimed to evaluate the effect of SSRIs on the expression of miRNAs and their protein targets.

**Materials and Methods:**

In a 100 day open-label study of citalopram (n=25) and sertraline (n=25), levels of miRNA 16, 132, and 124 and glucocorticoid receptor (GR), Brain-derived neurotrophic factor (BDNF), and serotonin transporter (SERT) protein expression were measured by QRT-PCR and western blot in healthy control (n=20), patients with depression at the baseline, and same patients after 100 days of treatment.

**Results:**

Expression levels of GR and BDNF proteins were lower in the depressed group before treatment as compared with the healthy group (*P<*0.0001). The SERT level was higher among the depressed group before treatment in comparison with the healthy group (*P<*0.0001). The level of GR and BDNF significantly increased, and SERT expression decreased after receiving sertraline (*P<*0.05). When the depressed group received citalopram, only SERT and GR were altered (*P<*0.05). Among the microRNAs’ expression investigated, mir-124 and mir-132 were higher, and mir-16 was lower among the depressed compared with the healthy group (*P<*0.0001). Individuals receiving citalopram only showed an increase in the expression of mir-16 while administration of sertraline led to a significant increase in the expression of mir-16 and a decrease in mir-124 and mir-132 (*P<*0.05).

**Conclusion:**

This elucidated the relationship between antidepressant treatment and the expression of different microRNA that control gene expression in various pathways involved in depressed patients. Receiving SSRI can affect the level of these proteins and their relevant microRNAs.

## Introduction

Depression is a prevalent psychiatric disorder with a considerable impact on global health. The prevalence of this disorder varies among populations, ranging from 17 to 53% of outpatients referring to medical departments (1). 

Approximately 50% of depression risk is recently contributed to genetic factors (2). Additionally, emerging evidence indicates that changes in gene expression play a crucial role in the pathogenesis of depression (3). Depressed patients have shown hyperactivity of the hypothalamus-pituitary-adrenal (HPA) and indeed increased cortisol levels (4). Cortisol has its effects by activating the glucocorticoid receptor (GR), which is also a regulator of further neurotrophic factors including the brain-derived neurotrophic factor (BDNF) (4). Therefore, the abnormal expression of this receptor can further result in the abnormal activity of the HPA axis and the induction of psychiatric manifestations, including depression (4). BDNF plays a crucial role in the regulation of neuronal cell growth, differentiation, and the pathophysiology of several mental disorders such as depression (5). Generally, BDNF levels of brain regions are lower in major depression disorder (MDD) patients, and antidepressant treatment may increase the BDNF level back to normal (5). Along with the relation between BDNF levels, the serotonin transporter (SERT) is another critical factor that is the target of antidepressant drugs, including selective serotonin reuptake inhibitors (SSRIs) that bind to SERT and inhibit serotonin reuptake (6). Moreover, a recent study reported the possible link between down-regulation of BDNF and SERT dysfunction in the prefrontal cortex and hippocampus (7). Likewise, reduced BDNF further affects GR and probably the HPA axis and thus causes psychiatric symptoms (8). 

Recently the contributing role of microRNAs as small non-coding RNAs has become an area of interest for many researchers. MicroRNAs can influence gene expression and induce up-regulation or down-regulation of different pathways involved in depression. Some microRNAs, including mir-132, are important in dendritic cell protruding, branching, and outgrowth as well as the development of hippocampal spine formation (9). In addition, mir-124, along with mir-132 is essential in synaptic plasticity and density (10). Moreover, mir-124 can be rapidly regulated by serotonin and lead to the enhancement of serotonin-dependent long-term facilitation (11). This microRNA, along with mir-132 plays an important role in the pathogenesis of psychiatric disorders such as depression and mood disorders (10, 12-14). Furthermore, these microRNAs are altered during stress and show modifications after antidepressant treatment (10). 

It has been well established that mir-16 can actively regulate SERT while mir-132 can modulate BDNF gene expression (15-17). Similarly, mir-124 plays a crucial role in maintaining the required GR expression levels for neurogenesis (10). Consequently, the levels of mir-16 as a post-transcriptional repressor of SERT, which is the target of SSRIs, may alter by following the antidepressant treatment (18). It has also been demonstrated that antidepressant therapy can affect both mir-124 and mir-132 levels in addition to the BDNF neurotransmitter (19). These results suggest that microRNAs may potentially be the biomarkers of brain function for depression and treatment response in depressed patients. Recently, some studies have reported the relations between specific genes and some microRNAs and depression rating scales although their results remain controversial (19, 20). 

Considering that measuring miRNAs or their target gene expression levels in brain tissues from patients is difficult, some researchers suggested that miRNAs can be secreted from cells into the peripheral tissue and plasma; therefore, brain-specific miRNAs in the blood can be direct indicators of the alterations survey in brain miRNA expression (17, 19, 21-26). Based on the findings of a study (2018), mir-124 and mir-132, which are tissue-specific and restricted to the nervous system, can travel for circulation in pathological and non-pathological conditions (19, 23). Moreover, studies demonstrated that BDNF and SERT in patients’ peripheral blood indicate a psychiatric disorder. Accordingly, their expression level measurement can be used to determine the patient’s response to antidepressant therapy (27, 28). 

Given the insufficient data on the relationship between antidepressant treatment and different microRNAs and their target gene expression in depressed patients, the present study aimed to evaluate the effect of SSRIs on the expression of miRNAs and their protein targets. To this end, mir-16, 132, and 124 were selected as critical miRNAs in depression, along with their target proteins including SERT, BDNF, and GR, respectively. 

## Materials and Methods

***Design of the study***

An open-label study was approved by the Ethics Review Board of Mashhad University of Medical Sciences, Iran (NO.IR.MUMS.SP.1395.8.). Fifty patients with moderate to severe depressive disorder who were referred to outpatient clinics of Ibn-Sina Psychiatry Rehabilitation Hospital (Mashhad, Iran) from January to February 2018 were considered as case and 20 healthy volunteers were considered as a control group. In addition, the study was registered at the Iranian Registry of Clinical Trials at http://www.irct.ir: IRCT20171112037407N1. This study is a continuation of our previous study, and some samples and information from the previous study have been used in this study. Therefore, the study design like case exclusion, dose of ingestion, calculation of sample size, and duration of treatment are similar to the previous study (29). According to a systematic review of SSRIs drug (30) and other references (31), some therapeutic properties, toxic effects and effects on the gene expression of types of SSRIs are different. Therefore, we selected two different drugs from this group for effect comparison. Due to the higher rate of sertraline and citalopram prescription by psychiatrists for the treatment of depression, they were selected among SSRIs (32, 33).

According to the inclusion criteria, the study participants were aged between 18 to 60 years. The depression score based on the Hamilton criteria was more than 17 for the patients (moderate and severe depression) and lower than 8 for the healthy volunteers. The exclusion criteria were as follows: history of any diseases requiring treatment by any drug for more than one month, hormone therapy, history of irritable bowel syndrome (IBS) and allergic diseases, history of antidepressants and use of central nervous system (CNS) agents during the last six months, history of bipolar disorder/psychosis, acute suicidality, ongoing electroconvulsive therapy (ECT), previous treatment with an SSRI, mental retardation, obsessive-compulsive disorder (OCD), anxiety, personality disorders, renal or liver disease, pregnancy, lactation, addiction, alcohol consumption, and smoking. Written consent was obtained from all subjects to participate in the study.

Diagnosis of depression was confirmed by interviews with psychiatrists based on the Diagnostic and Statistical Manual of Mental Disorders (DSM-IV). According to the psychiatrist’s prescription, 25 patients took citalopram (20 mg/day tablets, Abidi, Tehran, Iran), and 25 patients took sertraline (50 mg/day tablets, Sobhan Darou, Tehran, Iran). As titration, citalopram and sertraline were started with a single oral dose of 10 and 25 mg/day and then increased the dose to 20 and 50 mg/day in the second week, respectively. After 40 days, for patients who needed an increase in their medicine doses, double doses were prescribed. No patients received any other medication.

We collected blood samples from all participants (case and control groups) who fulfilled the exclusion and inclusion criteria at the baseline and from only patients after one hundred days of treatment by SSRIs. Each participant answered self-rated questionnaires to collect demographic data and HAM-D (Hamilton Depression Rating Scale) (Iranian state, HDRS21) (34) test to record their depression score before and after treatment by SSRI. Demographic data included age, gender, body mass index (BMI), occupation, marital status and education, history of smoking, alcohol consumption, medication, and disease, history of psychiatric disorders in the family, previous use/abuse of drugs, previous referral to psychiatrist and psychologist, and history of other mental disorders were recorded by a questionnaire designed for this study. We followed up with patients on the 40^th^, 70^th^, and 100^th^ days of treatment by interviewing them where the incidence of adverse effects and other diseases was recorded. 

*Sample size*

According to the investigation of Stephen M. Stahl (35), we assumed the minimum detectable difference in the means of 14.00 in HAM-D scores changes, the standard deviation of 10.00, Number of 3 Groups, power of 0.90, and a significance level of 0.05 (Alpha). Then, a minimum sample size of 14 participants in each group was calculated by SigmaPlot version 12.0 (Systat Software, Inc., Germany) which was applied for sample size calculation.

***Blood sample collection***

We have detected the change in GR, SERT, and BDNF protein expression in leukocytes. The miRNAs were evaluated in the plasma of patients since they can be secreted from brain cells into the plasma (36). For the detection of protein expression, 5 ml of blood was obtained in a heparin tube by venipuncture (Venoject) from each participant. On the day of the venipuncture, peripheral blood mononuclear cells (PBMC) were isolated from whole blood by lymphocyte separation medium (Lymphosep, Biowest, France). Leucocytes were cryopreserved in a freezing mix (90% v/v Fetal Bovine Serum (FBS) and 10% v/v DMSO). Also, for determination of miRNAs expression in the circulation system, 3 ml of whole blood was collected from each participant in EDTA tubes, and plasma was isolated by immediate centrifugation at 3000 rpm for 15 min at 4 ^°^C. The leucocytes and plasma were then aliquoted and stored at -80 ^°^C prior to analysis by western blotting and quantitative real-time polymerase chain reaction (QRT-PCR).

***Experiments***

*QRT-PCR*

miRNA was extracted from plasma by miRNeasy Serum/Plasma kit (Qiagen, USA). Then, miRNA concentrations and purity (A260/A280) were measured by NanoDrop^TM ^2000/2000c spectrophotometers (Thermo Fisher Scientific, USA). The miRNA was converted to the first-strand complementary DNA (cDNA) with RT stem-loop miRNA and HK (housekeeping) primers provided by Zist Royesh cDNA synthesis kit (Tehran, Iran). It was frozen at -80 °C until quantitative real-time polymerase chain reaction (QRT-PCR) was performed. The miRNA expression levels of human mir-16, 124, and 132 were measured in the samples of patients with depression (N=50) and healthy control (N=20) groups at the baseline and after 100 days only for the treatment group (N=50). For measuring mir-16, 124, and 132 expressions, QRT-PCR was performed using the SYBR GREEN qPCR master mix kit (RealQ Plus Master Mix Green, Amplicon, Stenhuggervej, Denmark). The amplifications were done in Light Cycler 1.5 (Roche Applied Science, Penzberg, Germany). U6 [is a small nuclear RNA (snRNA) which is highly conserved among species and is one of the most widely used internal reference genes for miRNA] was employed as a housekeeping gene for normalizing the differences of the cycle of threshold (Ct) between the reference and target genes (ΔCt) (37). The ΔΔCt ratio was calculated based on the differences between the ΔCt sample and ΔCt reference. Finally, ΔΔCt was used to analyze the gene expression levels (fold change (FC) of expression) by formula 2^–^^ΔΔCt^. Three independently prepared samples were used for each data point. All of the experiments were performed in triplicate. The HK-specific (U6) and miRNA-specific (miR-132, 124, and 16) forward and reverse primers were synthesized by Metabion International AG (Germany). The coefficient of variations (% CV) for the Ct of mir-16, 124, 132, and U6 were 0.63, 1.1, 0.94, and 0.87, respectively.

*Western blot*

After the thawing of cryopreserved leucocytes, the cells were centrifuged at 3000 rpm for 15 min. Then, the cell pellet was re-suspended in a lysis buffer containing 10 mM HEPES, 1.5 mM MgCl_2_, 10 mM KCL, 0.5 Mm DDT, 0.05% NP40 (pH=7.6), and phosphatase inhibitor cocktail. After incubation on ice for 10 min, the homogenates were centrifuged at 12000 rpm for 2 min at 4 ^°^C, and the cytoplasmic extract was carefully removed from the nuclear pellet. In the next step, nuclear extraction buffer containing 5 mM HEPES, 1.5 mM MgCl_2_, 0.2 mM EDTA, 26% Glycerol, 0.5 Mm DDT, 4.6 M NaCl, and phosphatase inhibitor cocktail in pH of 7.6 was added to the nuclear pellet. The extract was incubated on ice for 15 min with vortexing (5 sec) every 3 min. The extract was then sonicated for 30 sec to increase nuclear protein extraction. The suspension was centrifuged for 20 min at 14,000 rpm, 4 ^°^C, and the supernatant was collected. The total protein concentration of cell extract was detected by a Pierce™ BCA Protein Assay Kit from Thermo Fisher Scientific. 

An equal amount of proteins were separated by SDS-PAGE and transferred to a PVDF membrane. After the membranes were blocked in 5% dried skimmed milk in TBST buffer (Tris-buffered saline, 0.1% Tween 20) at room temperature for two hours, incubation with the primary antibodies was performed for a further 2 hr at room temperature (Rabbit monoclonal anti-serum against GR (3671): 1:1000, Rabbit monoclonal anti-serum against SERT (2662): 1:1000 and Rabbit monoclonal anti-serum against BDNF (108319): 1:1000, from Abcam, USA, and Mouse monoclonal anti-serum against β-Actin (3700): 1:2000 as an internal control for normalizing from Cell Signaling, USA. Then, membranes were washed three times with TBST before incubation with an Anti-rabbit IgG with horseradish peroxidase (HRP: 7074), and Anti-mouse IgG with horseradish peroxidase (HRP: 7076) labeled secondary antibody (1:3000). Then, the membrane was rewashed three times by TBST. Finally, the immunoreactive bands were visualized using the enhanced chemiluminescence (Luminol and p-Coumaric acid from Sigma-Aldrich, USA) method, Gel Doc (gel documentation system, UVItec, United Kingdom), and UV band software. The results were normalized using β-Actin expression as the internal standard. All of the experiments were performed in triplicate and averages of the results of triplicate tests were used.

***Statistical analysis***

For multiple comparisons, the results were statistically analyzed using SPSS version 22 and GraphPad Prism 7. The normality of the group was tested by Shapiro-Wilk Test and graphic method (histogram curve). Appropriate parametric and non-parametric tests were used. Differences between groups or two-tailed tests with a *P*-value less than 0.05 were statistically considered significant. Descriptive analysis for parametric data is expressed as mean value±standard deviation (SD), and non-parametric data are expressed as median (IQR). At the baseline, demographic data between the healthy control, citalopram, and sertraline groups were compared by the One Way ANOVA test. The quantitative variable of parametric data between two groups was compared using an independent sample t-test or pair t-test, and non-parametric was analyzed using Man Whitney or Wilcoxon test. Pearson’s and spearman’s correlation coefficient was used to examine the strength of linear association between two variables of parametric data and non-parametric data, respectively.

## Results

***Clinical characteristics of*** ***participants and demographic information ***

The total of 50 patients was divided into citalopram (n=25) and sertraline (n=25) groups, and 20 healthy volunteers as a healthy control group enrolled in the study. The number of participants, gender, age, BMI, and HAM-D scores (depression score) in the healthy control, citalopram, and sertraline groups (at the baseline) are shown in Table 1. Mean age and BMI among control and patient groups were not significantly different. Before treatment (BT), demographic data including age, gender, BMI, and HAM-D scores were not significantly different between the citalopram and sertraline groups. After treatment (AT) with SSRIs, means±SD of HAM-D scores were significantly decreased in citalopram (20.12±11.72) and sertraline (18.05±11.74) groups. There was no significant difference in the level of reduction of the HAM-D score between the citalopram and sertraline groups (*P*=0.77). Furthermore, following 100 days of treatment, significant changes in BMI were not observed in either group (*P*=0.24).

***Levels of protein expression***

SERT, BDNF, and GR protein expression levels were analyzed using western blot in healthy control and depressed groups at the baseline, and in the depressed group after treatment with sertraline and citalopram (in separate group).

**Figure 1:**
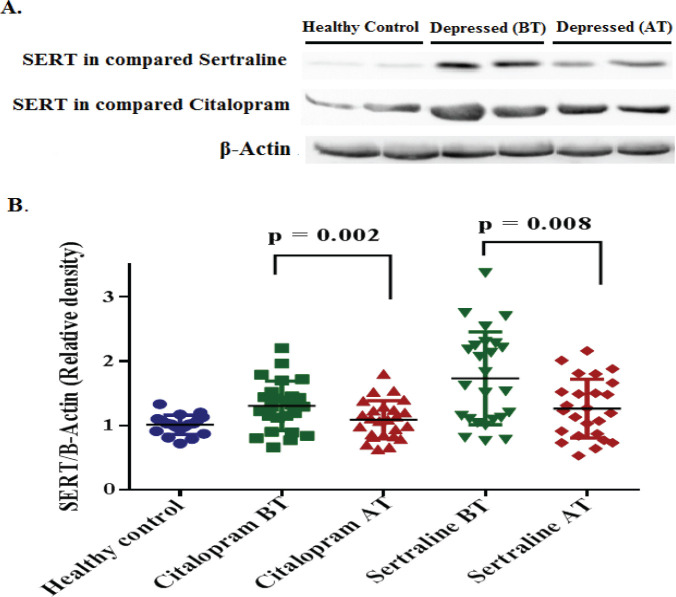
Expression of SERT protein in PBMCs of participants

**Figure 2:**
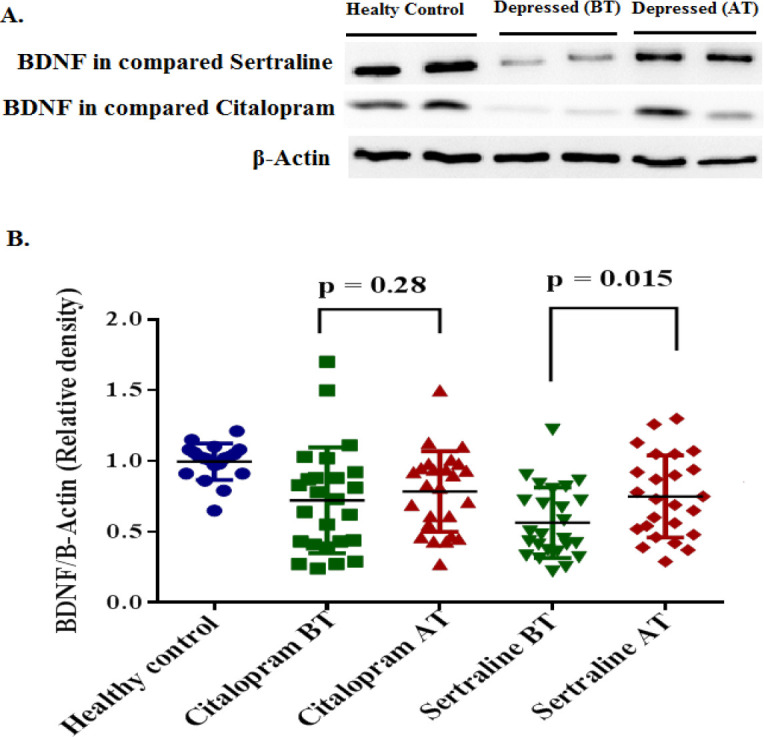
Expression of BDNF protein in PBMCs of participants

**Figure 3:**
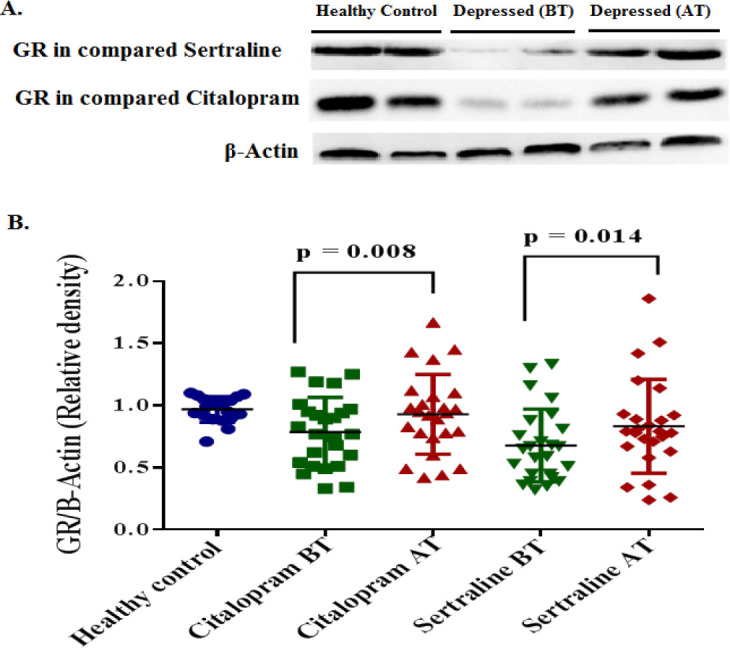
Expression of GR protein in PBMCs of participants

At the baseline, means±SD of the relative optical density of SERT proteins were significantly lower in the healthy control group as compared with case groups (*P*=0.0007). However, average relative BDNF and GR expression levels were significantly lower in the depressed group in comparison with the control group *P*<0.0001 and *P*=0.0009, respectively. After treatment with SSRIs, the average SERT level was significantly decreased in both citalopram (*P*=0.002) and sertraline (*P*=0.008) groups (Figure 1). While, the mean±SD of BDNF expression was significantly increased in the sertraline group (*P*=0.015). There was no significant difference between the citalopram group compared with baseline. (*P*=0.28) (Figure 2). Additionally, the expression level of GR was significantly increased following treatment with citalopram (*P*=0.008) and sertraline (*P*=0.014) (Figure 3). No significant difference in efficacy between sertraline and citalopram was found as related to the expression of SERT (*P*=0.151), BDNF (*P*=0.181), and GR (*P*=0.871).

***Levels of miRNA expression***

We investigated expression levels of mir-16, 124, and 132 using real-time PCR in healthy control and depressed groups at the baseline, and following treatment with sertraline and citalopram in a separate group.

*mir-16*

**Figure 4:**
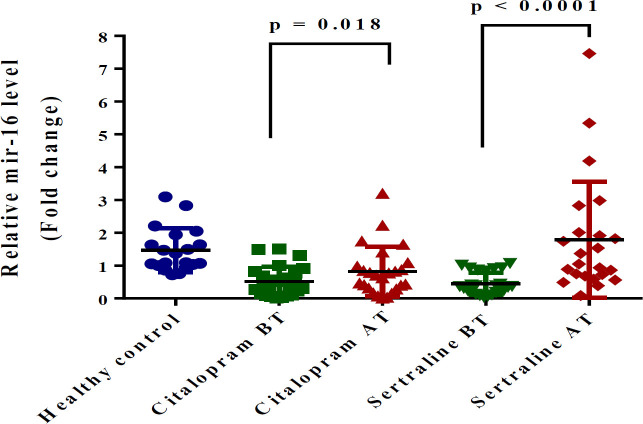
Graphical representation of fold change (FC=2-^ΔΔCt^) of mir-16 expression

At the baseline, mir-16 expression in patients was significantly lower than the expression level in all patients as compared with the healthy controls (*P*<0.0001). Since, Shapiro-Wilk test proved abnormal distribution of mir-16 in citalopram (citalopram BT: *P*=0.01, citalopram AT: *P*=0.002) and sertraline groups (sertraline BT: *P*=0.002, sertraline AT: *P*<0.001), we used nonparametric tests for comparison. After treatment by SSRIs, the median (IQR) of mir-16 expression was significantly increased in citalopram (*P*=0.041) and sertraline (*P*=0.024) groups (Figure 4). We found a significant difference in the increase of mir-16 expression between citalopram and sertraline groups (Mann Whitney U test, *P*=0.03). Consequently, the efficacy of sertraline in increasing mir-16 was greater than citalopram.

*mir-132*

**Figure 5:**
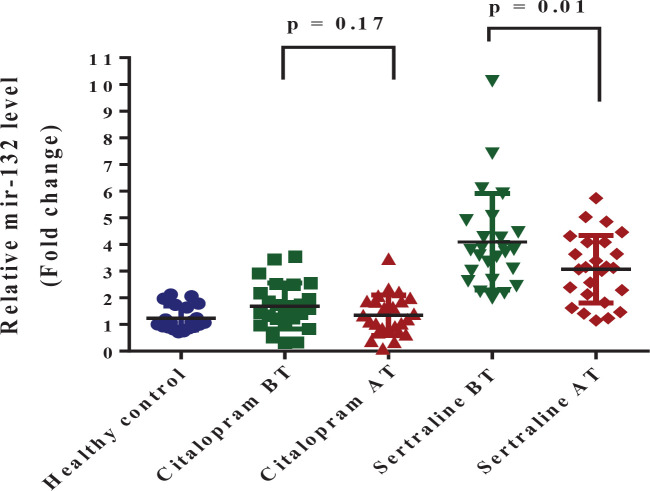
Graphical representation of fold change (FC=2-^ΔΔCt^) of mir-132 expression. Comparison of mir-132 expression levels (Means±SD) between the control (n=20) and patient (n=50) groups at the baseline (independent samples T-Test, *P<*0.0001)

Before treatment, the miR-132 expression was significantly higher in the depressive group than in the healthy control group (*P*=0.0003). After treatment by SSRIs, the mean±SD of miR-132 expression in the sertraline group was significantly lower than before treatment (*P*=0.011). But, in citalopram groups, there was no significant difference in the expression of mir-132 levels before and after treatment (*P*=0.17) (Figure 5). No difference was found in efficacy between sertraline and citalopram in mir-132 expression (*P*=0.142).

*mir-124*

**Figure 6:**
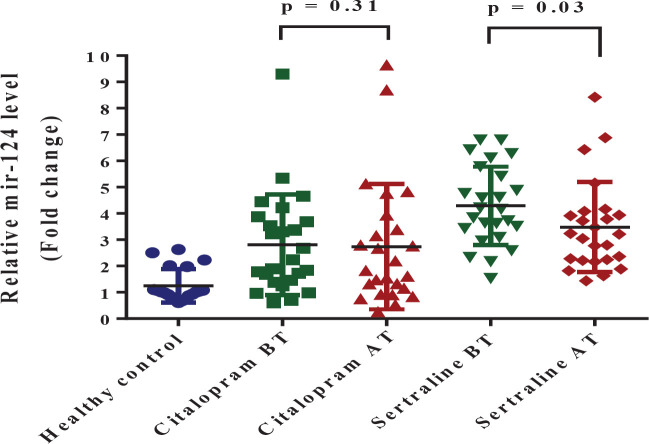
Graphical representation of fold change (FC=2- ^ΔΔCt^) of mir-124 expression. Comparison of mir-124 expression levels (Median score (IQR)) between the control (n=20) and patient (n=50) groups at the baseline (Mann-Whitney U test, *P<*0.0001)

The median (IQR) of mir-124 levels in the patients was much higher than in the healthy control group (*P*<0.0001) before treatment. As Shapiro-Wilk Test proved abnormal distribution of mir-124 in citalopram (citalopram BT: *P*=0.002, citalopram AT: *P*<0.001) and sertraline groups (sertraline BT: *P*=0.53, sertraline AT: *P*<0.001), we used non-parametric tests for comparison. After treatment with SSRIs, miR-124 expression of the sertraline group was significantly lower than before treatment (*P*=0.03). While, among citalopram groups, there was no statistical difference in mir-124 levels after treatment compared with baseline. (*P*=0.31) (Figure 6). No difference was found in efficacy between sertraline and citalopram in mir-124 expression (*P*=0.163).

***Correlation between HAM-D and miRNAs and protein expressions***

In the patients, we did not find any correlation between HAM-D scores and miRNA levels or HAM-D and protein expression, at the baseline. Also, data on miRNA levels were not associated with protein expression levels. On the other hand, there was no correlation between HAM-D scores, miRNAs, and protein expression levels concerning the rate of change after treatment. 

## Discussion

**Figure 7:**
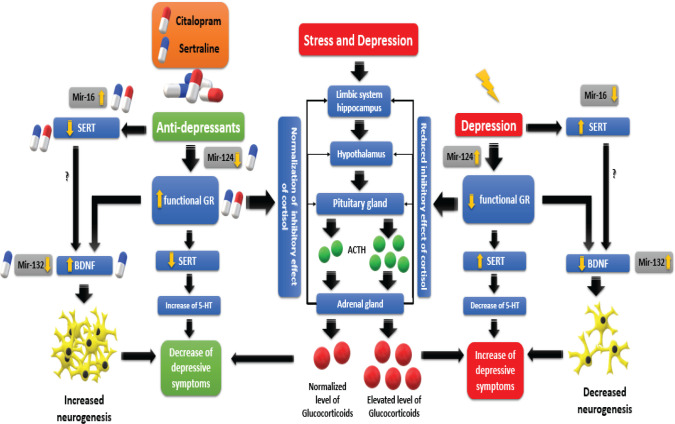
Image of summary of our results (all changes directly found in this study were indicated by the yellow arrow - decrease or increase ) and depiction of the possible mechanistic pathway of depression and its treatment by citalopram and sertraline on some miRNA and protein expressions. In this study, sertraline ( ) could cause a significant change in SERT, BDNF, GR, mir-16, mir-124, and mir-132 levels. But, citalopram ( ) changed the level of SERT, GR, and mir-16, significantly

The finding of the present study revealed that the expression level of SERT in depressed patients was higher compared with the healthy group while the expression levels of GR and BDNF proteins were lower in the depressed group in comparison with the healthy group. Based on the results, the level of SERT, GR, and BDNF significantly altered after receiving sertraline while receiving citalopram could only alter SERT and GR. All the investigated microRNAs in this study differed in terms of miRNA expression among depressed and healthy groups. When evaluating the therapeutic effect of antidepressants, those receiving citalopram only showed altered miR-16 expression while patients who received sertraline represented altered expression in all three microRNAs (Figure 7). 

The findings further revealed a decrease in the GR level in depressed compared with healthy individuals. The reduced inhibitory effect of cortisol on upstream glands also reduces during stress or psychological conditions including depression, and the overproduction of *adrenocorticotropic hormone* results in increasing circulatory glucocorticoids (38). According to a study, impaired GR can lead to impaired feedback inhibition of cortisol and, therefore, the hyperactivation of the HPA axis (Figure 7) (4). Considering that GR is a regulator of BDNF, which can alter hippocampal neurogenesis (39), a decreased BDNF level may result in a reduced number of brain neurons whereas an increased level may lead to active neurogenesis (8, 40). In the study by Bocchio-Chiavetto *et al*. (17), miRNAs revealed a critical role in modulating neurogenesis and synaptic plasticity, especially for the pathophysiology of psychiatric disorders and psychotropic drugs’ effectiveness. Additionally, these can affect the regulation of axonal and dendritic outgrowth or morphology and the homeostasis of neurotransmitters (17, 41-43).

The BDNF expression level varies in different areas of the brain (44). Contrary to the hippocampus, an increase in the level of BDNF in the basolateral amygdala and amygdala can induce anxiety (44). Regardless of the BDNF level in different areas of the brain, the peripheral BDNF level is correlated with depression and the treatment response (20, 45). Researchers reported in their meta-analysis that peripheral BDNF levels decreased in MDD patients and accounted for the BDNF level as a biomarker rather than a risk factor of MDD (20, 45). The present study only evaluated the systemic level of these proteins in circulation. Similar to the results of the above-mentioned meta-analysis, those of the current study demonstrated that depressed patients had decreased peripheral BDNF levels, and a significant increase of BDNF and GR levels was observed in sertraline-treated patients. Moreover, SERT plasma distribution and total expression can change according to specific conditions, resulting in psychiatric changes during life (46). Based on the findings of the present study, the SERT level significantly differed among depressed and healthy controls, which is in line with the results of another study, representing that the SERT mRNA level increased in depressed patients while it decreased after SSRI treatment (47). However, in another study with greater sample sizes and more prolonged treatment periods, Kao *et al*. found that SERT mRNA increases after antidepressant treatment (48). Recently, researchers have addressed the possible relation between SERT or serotonin signaling and BDNF by studying mice models and reported that a complete lack of brain serotonin induces an increase in BDNF expression although SERT-deficient mice did not show such changes (49). Conversely, a study found different findings, showing that the BDNF level significantly decreases in the hippocampal and prefrontal cortex of SERT knock-out rats (50). In contrast, no significant association was observed between SERT and BDNF changes in each individual in the present study. However, a decrease in the expression level of BDNF in patients with depression was generally accompanied by increased SERT (50). 

Depressed patients can benefit from various pharmaceutical and non-pharmaceutical treatments (51). The present research focused on two well-known antidepressant agents, including citalopram and sertraline from the SSRI category that act selectively on serotonin receptors (52). According to our results, both of these drugs could affect GR and SERT serum levels while only sertraline could significantly increase the BDNF level. Additionally, Balu *et al*. revealed that administration of antidepressants and antipsychotics could increase BDNF protein in the frontal cortex (53). A recent meta-analysis demonstrated that antidepressant therapy could increase the BDNF level and this effect largely relied on the antidepressant type (54). In agreement with this meta-analysis, our findings suggested that sertraline can increase the BDNF level in a short time while citalopram failed to provide such an effect. A possible explanation of the insignificant effect of citalopram on the BDNF level could be the time effect. The long-term follow-up of the depressed patient’s treatment may change the results, and this explanation needs further studies with larger sample sizes and different times of the therapeutic duration (55). 

Another study showed that sertraline and paroxetine could decrease the SERT density and even its function (56). Moreover, chronic treatment by SSRIs, including fluoxetine, increases the BDNF level and GR phosphorylation in BDNF-responding sites and results in a further increase in GR-dependent hippocampal neurogenesis (57). Although there are insufficient clinical data on antidepressant effects on GR, BDNF, or SERT levels, their possible impacts can be summarized according to our results and the available literature on cellular and animal studies. Both sertraline and citalopram may restore the dysregulated HPA axis and reestablish the dysregulation of the HPA axis. In addition, they cause a decrease in the GR level while increasing the serotonin level by blocking their reuptake. 

The findings of the current study represented that changes in the expression in each of the three proteins were accompanied by expression changes in the three microRNAs. Additionally, the results revealed that in depression, an increase in mir-132 and 124 levels are accompanied by a reduction in BDNF and GR levels, respectively. Furthermore, a decrease in the mir-16 level was associated with the increasing level of SERT. During stress, some microRNAs, including mir-124 and mir-132, show altered expression in different areas of the brain (10). In general, both of these microRNAs are important in neuronal maturation, and each has its unique downstream targets. For instance, mir-132 has a regulatory effect on CREB (58, 59) while, CREB affects BDNF, which can further inhibit mir-132. On the other hand, mir-124 can inhibit CREB and GR (20, 60). The results of the present study showed that the level of mir-124 increases in depression. Although no significant correlation was observed between mir-124 and GR levels in this study, the GR level decreased with an increase in mir-124 (Figures 3 and 6). Overall, the cortisol level will increase during depression and stress and thus the body increases the level of mir-124 (60). Furthermore, mir-124 is considered an inhibitor of GR, therefore, the GR level decreases by the regulatory effect of mir-124. The same inverse relationship can be interpreted from the mir-132 and BDNF relation in depression disorder (Figures 2 and 5). 

According to the results of the current study, mir-132 and 124 can be differently influenced in response to various SSRIs and only sertraline can change mir-132 and 124 expressions. Contrarily, Fang *et al*. found that MDD patients receiving no treatment had significantly higher mir-132 levels compared with those who received citalopram (19). Furthermore, both their treated and untreated MDD patients had higher BDNF levels in comparison with the healthy group although the BDNF level was not significantly different between the patients of both groups (19). 

Accompanying our results, it has been shown that the neutralization of mir-16 could increase SERT expression and the efficacy of SSRIs (18). In addition, mice receiving chronic antidepressant treatment show an increased level of mir-16 in raphe and, as a result, reduced SERT level (61). In the current study, both citalopram and sertraline can increase mir-16 expression, while the efficacy of sertraline in increasing mir-16 was greater than citalopram. In the hippocampus, fluoxetine decreases the mir-16 level while increasing the SERT level and sustaining hippocampal neurogenesis (18). Moreover, research demonstrated that the mir-16 level significantly increases in responders after treatment with antidepressants (62), which corroborates with the results of the present study. Similarly, Song *et al*. evaluated human subjects with MDD and revealed that the cerebrospinal fluid mir-16 was lower in these patients compared with healthy subjects (62). However, the blood level of this microRNA was not significantly different among depressed and healthy subjects (63), which contradicts the findings of the current study. 

Depression can be rated by using different questionnaires and rating scales including HAMD (64). In the present study, no significant relation was found between the results of this rating scale and those of each of the examined microRNAs and protein changes. In contrast to the current research, a study reported a positive correlation between miR-132 and HAMD scores (19). However, there was no significant relation between HAMD scores and BDNF and mir-124 levels (19), which matches the findings of the present study. Another study showed that the BDNF level is correlated with another depression rating score (20). They further revealed a significant negative correlation between the self-rating depression scale (SDS) score and BDNF levels while showing a positive correlation between the SDS score and the serum mir-132 level (20). Moreover, they found a reverse relation between the BDNF level and mir-132 and mir-182 levels in depressed patients (20). In contrast to our study, Song *et al*. demonstrated that mir-16 is negatively correlated with Hamilton scores (63). 

The present study had some limitations. The levels of proteins and microRNAs were only evaluated in lymphocytes and plasma but not in other body fluids such as cerebrospinal fluid or different parts of the brain. It is well established that the level of these proteins and their corresponding microRNAs differs in various areas of the brain. In addition, the small treatment period and sample size may have affected the results. Furthermore, the lack of a control group at 100 days could be considered a limitation. Finally, polymorphisms and other genetic variations in each of the patients were other limitations, which have not been evaluated in this study. Various pathways are enrolled in the treatment response to antidepressants, and the present study only assessed the three main proteins in such pathways. 

A: Representative Western blot image for SERT and β-Actin (as normalizer) proteins; one case of the control group (Healthy control); b: One of the patients with depression disorder before treatment (Depressed (BT)); same patient after 100 days of SSRI treatment (Depressed (AT)); B: Graphical representation of the average expression intensity of SERT protein relative to β-Actin protein. Comparison of SERT expression levels (Mean±SD) between the control (n=20) and patient (n=50) groups at the baseline (independent samples T-Test, *P<*0.0001). Comparison of SERT expression levels (Mean±SD) between patients in the citalopram group (n=25) at the baseline (Citalopram AT) (1.3±0.38) and after treatment (Citalopram BT) (1.08±0.29) (Pair T-Test, *P=*0.002) and between patients in sertraline group (n=25) at the baseline (Sertraline AT) (1.73±0.72) and after treatment (Sertraline BT) (1.26±0.45) (Paired T-Test, *P=*0.008)

**Table 1.** Demographic data of the healthy control and patient groups at the baseline (one way ANOVA)

	**Healthy control group**	**Citalopram group**	**Sertraline group**	***P*** **-Value**
**Number (male, female)**	20 (10, 10)	25 (10, 15)	25 (7, 18)	0.20
**Age ** **(Mean** ** ± SD)**	30.2± 4.69	35.43 **± **6.76	36.17 **± **8.02	0.33
**BMI ** **(Mean** ** ± SD)**	21.32 **± **5.71	26.3 ± 3.95	26.76 ± 4.54	0.60
**HAM-D ** **Scores (Mean** ** ± SD)**	4.17 ± 1.02	28.08 ± 7.52	27.55 ± 10.68	0.77*

* This data was gained from the analysis between the citalopram and sertraline groups by independent T-Test

A: Representative western blot image for BDNF and β-Actin (as normalizer) proteins; one case of the control group (Healthy control); b: One of the patients with depression disorder before treatment (Depressed (BT)); same patient after 100 days of SSRI treatment (Depressed (AT)); B: Graphical representation of the average expression intensity of BDNF protein relative to β-Actin protein. Comparison of BDNF expression levels (Mean±SD) between the control (n=20) and patient (n=50) groups at the baseline (independent samples T-Test, *P<*0.0001). Comparison of BDNF expression levels (Mean±SD) between patients in the citalopram group (n=25) at the baseline (Citalopram BT) (0.72±0.37) and after treatment (Citalopram AT) (0.78±0.28) (Paired T-Test, *P=*0.28) and between patients in sertraline group (n=25) at the baseline (Sertraline BT) (0.56±0.24) and after treatment (Sertraline AT) (0.74±0.28) (Paired T-Test, *P=*0.015)

A: Representative western blot image for GR and β-Actin (as normalizer) proteins; one case of the control group (Healthy control); b: One of the patients with depression disorder before treatment (Depressed (BT)); same patient after 100 days of SSRI treatment (Depressed (AT)); B: Graphical representation of the average expression intensity of GR protein relative to β-Actin protein. Comparison of GR expression levels (Mean±SD) between the control (n=20) and patient (n=50) groups at the baseline (independent samples T-Test, *P<*0.0001). Comparison of GR expression levels (Mean ± SD) between patients in the citalopram group (n=25) at the baseline (Citalopram BT) (0.78±0.27) and after treatment (Citalopram AT) (0.92±0.32) (Paired T-Test, *P=*0.008) and between patients in sertraline group (n=25) at the baseline (Sertraline BT) (0.67±0.29) and after treatment (Sertraline AT) (0.83±0.37) (Paired T-Test, *P=*0.014)

Comparison of mir-16 expression levels (Median score (IQR)) between the control (n=20) and patient (n=50) groups at the baseline (Mann-Whitney U test, *P<*0.0001). Comparison of mir-16 expression levels (Median score (IQR)) between patients in the citalopram group (n=25) at the baseline (Citalopram BT) (0.39 (0.66)) and after 100 days of treatment (Citalopram AT) (0.71 (0.73)) (Wilcoxon test, *P=*0.018) and between patients in sertraline group (n=25) at the baseline (Sertraline BT) (0.36 (0.64)) and after 100 days of treatment (Sertraline AT) (1.37 (2.2)) (Wilcoxon test, *P<*0.0001). ΔCt: difference of Ct between the target gene and the U6 (housekeeping) gene. Normality was assessed by Shapiro-Wilk normality tests

IQR: Inter quartile range

Comparison of mir-132 expression levels (Means±SD) between patients in the citalopram group (n=25) at the baseline (Citalopram BT) (1.69±0.86) and after 100 days of treatment (Citalopram AT) (1.34±0.76) (Paired t-Test, *P=*0.17) and between patients in sertraline group (n=25) at the baseline (Sertraline BT) (4.09±1.81) and after 100 days of treatment (Sertraline AT) (3.07±1.27) (Paired t-Test, *P=*0.01). ΔCt: the difference of Ct between the target gene and the U6 (housekeeping) gene. Normality was assessed by Shapiro-Wilk normality tests

Comparison of mir-124 expression levels (Median score (IQR)) between patients in the citalopram group (n=25) at the baseline (Citalopram BT) (2.23 (2.36)) and after 100 days of treatment (Citalopram AT) (1.83 (2.62)) (Wilcoxon test, *P=*0.31) and between patients in sertraline group (n=25) at the baseline (Sertraline BT) (4.16 (2.33)) and after 100 days of treatment (Sertraline AT) (3.21 (1.78)) (Wilcoxon test, *P=*0.03). ΔCt: difference of Ct between the target gene and the U6 (housekeeping) gene. Normality was assessed by Shapiro-Wilk normality tests

IQR: Inter quartile range

## Conclusion

To the best of our knowledge, the present study is the first clinical study with restricted criteria that reveal that the protein levels of SERT, GR, and BDNF, as well as their corresponding microRNAs (i.e., 16, 124, and 132), differ between depressed and healthy individuals. Although no significant correlation was found between mir-124 and GR levels, a reduction in the GR level was inversely correlated with an increase in mir-124 expression. The same inverse relationship could be interpreted from mir-132 and BDNF relations in addition to mir-16 and SERT in depression disorder. This is probably because BDNF, GR, and SERT are regulated by many miRNAs and multiple factors, affecting the correlation between miRNAs and targets*. *Finally, receiving SSRIs could affect the level of these proteins and their corresponding microRNAs. 

## Authors’ Contributions

MA, MHGH, and MRA studied concepts and designed the research. MA, LE, and DMR performed data processing, collection, and experiment. PJ, SAM, and AHM performed analysis and interpretation of results. FB is a psychiatrist and helped in sampling. RJE and MA prepared the draft manuscript. MRA, SAM, PJ, AHM, and MHGH revised the manuscript. All authors approved the final version to be published. MRA supervised, and funding acquisition was done by SAM.

## Conflicts of Interest

The authors declare that they have no conflicts of interest.
